# Supplementary material for: The structure and diversity of freshwater diatom assemblages from Franz Josef Land Archipelago: a northern outpost for freshwater diatoms
Source: PeerJ. 2016 Feb 18;4:e1705. doi: 10.7717/peerj.1705 (PMC4768701; doi:10.7717/peerj.1705)
Supplement: Appendix S1 — Common diatoms species (abundance >2% at least in one sample) in FJL with the code used in plots, their geographic distribution, the mean, the maximum and the standard deviation of their relative abundance (percentages) in FJL samples. [file peerj-04-1705-s001.docx]

| Taxon Name | code | Distribution | mean | max | sd |
| --- | --- | --- | --- | --- | --- |
| *Diatoma problematica* Lange-Bertalot | DAproble | Widely | 4.51 | 80.40 | 18.94 |
| *Diatoma tenuis* Agardh | DAten | Widely | 12.42 | 49.00 | 15.08 |
| *Fragilaria capucina* (complex) Desmazières | FRcapcpx | Widely | 7.34 | 39.60 | 10.20 |
| *Rossithidium petersennii* (Hustedt) Round et Bukhtiyarova | RSpeters | Widely | 7.05 | 38.80 | 10.11 |
| *Eucocconeis laevis* (Østrup) Lange-Bertalot | ESlaevis | Widely | 3.06 | 37.40 | 9.43 |
| *Nitzschia alpina* Hustedt | NIalpina | Widely | 4.13 | 31.20 | 8.93 |
| *Psammothidium marginulatum* (Grunow)Bukhtiyarova et Round | PSmargin | Widely | 2.47 | 24.00 | 5.69 |
| *Tabellaria flocculosa* (Roth) Kützing | TAfloccu | Widely | 1.67 | 23.80 | 5.59 |
| *Nitzschia homburgiensis* Lange-Bertalot | NIhombur | Widely | 2.28 | 23.60 | 5.58 |
| *Diadesmis gallica* Smith | DIgallic | Widely | 2.06 | 22.60 | 5.63 |
| *Chamaepinnularia gandrupii* (Petersen) Lange-Bertalot et Krammer | CQgandru | Circumpolar | 4.65 | 21.60 | 7.26 |
| *Nitzschia perminuta* (Grunow) Peragallo | NIpermin | Widely | 7.96 | 21.40 | 7.08 |
| *Encyonema fogedii* [complex] Krammer | ECfogcpx | Unknown | 3.14 | 19.20 | 6.00 |
| *Fragilaria cf. perminuta* (Grunow) Lange-Bertalot | FRpermcf | Widely | 2.00 | 17.00 | 4.50 |
| *Meridion circulare* (Greville) Agardh | MDcircul | Widely | 1.16 | 16.00 | 3.74 |
| *Achnanthidium minutissimum* (Kützing) Czarnecki | AHminuti | Widely | 1.27 | 15.60 | 3.62 |
| *Hygropetra balfouriana* Grunow ex Cleve | HGbalfou | Widely | 3.02 | 15.00 | 5.17 |
| *Encyonema silesiacum* (Bleisch) Mann | ECsilesi | Widely | 0.88 | 14.80 | 3.48 |
| *Naviculadicta digituloides* Lange-Bertalot | NVdigdes | Widely | 0.77 | 12.60 | 2.96 |
| *Cymbella botellus* (Lagerstedt) Schmidt | CMbotell | Circumpolar | 0.83 | 11.00 | 2.59 |
| *Psammothidium kryophilum* (Petersen) E. Reichardt | PSkryoph | Widely | 1.52 | 10.80 | 3.07 |
| *Psammothidium sp1* (in Antoniades, 2009) | PSsp1ant | Circumpolar | 1.26 | 9.40 | 2.87 |
| *Nitzschia cf. sublinearis* Hustedt | NIsublcf | Circumpolar | 1.04 | 9.40 | 2.93 |
| *Hippodonta arctica* (Patrick & Freese)Lange-Bertalot, Metzeltin & Witkowski | HIarctic | Circumpolar | 0.56 | 9.34 | 2.19 |
| *Hannaea arcus* (Ehrenberg) Patrick | HNarcus | Widely | 0.61 | 9.20 | 2.15 |
| *Cymbella cleve-eulerae* Krammer | CMcleveu | Circumpolar | 0.69 | 8.60 | 2.08 |
| *Denticula tenuis* Kützing | DEtenuis | Widely | 0.44 | 7.80 | 1.84 |
| *Nitzschia acidoclinata* Lange-Bertalot | NIacidcn | Widely | 0.94 | 7.60 | 2.26 |
| *Nitzschia* spA | NIsp_A | Unknown | 0.69 | 7.40 | 2.02 |
| *Psammothidium helveticum* (Hustedt) Bukhtiyarova et Round | PShelvet | Widely | 0.51 | 7.40 | 1.74 |
| *Staurosirella pinnata* (Ehrenberg) Williams et Round | SApinnat | Widely | 0.70 | 7.00 | 1.96 |
| *Cymbopleura stauroneiformis* (Lagerstedt) K.Krammer | CBstauro | Circumpolar | 0.80 | 5.20 | 1.64 |
| *Humidophila laevissima* (P.T.Cleve) Lowe, Kociolek, Johansen, Van de Vijver, Lange-Bertalot & Kopalová | DIlaevis | Circumpolar | 0.64 | 5.20 | 1.34 |
| *Parlibellus protracta (Grunow) Witkowski, Lange-Bertalot et Metzeltin* | PBprotr | Widely | 0.28 | 5.12 | 1.21 |
| *Diadesmis* spA | DIsp_A | Circumpolar | 0.30 | 5.00 | 1.18 |
| *Humidophila ingeiiformis* (P.B.Hamilton & D.Antoniades) Lowe, Kociolek*,* Johansen, Van de Vijver, Lange-Bertalot & Kopalová | DIingicf | Circumpolar | 0.70 | 4.60 | 1.18 |
| *Planothidium peragalloi* (J.Brun & Héribaud-Joseph) Round & L.Bukhtiyarova | PTperaga | Widely | 0.36 | 4.20 | 1.09 |
| *Navicula* spB | NAsp_B | Unknown | 0.22 | 3.92 | 0.92 |
| *Psammothidium* grischunum (Wuthrich) Bukhtiyarova et Round | PSgrisch | Widely | 0.27 | 3.80 | 0.89 |
| *Gomphonema lapponicum* (A.Cleve) Cleve-Euler | GOlappon | Circumpolar | 0.24 | 3.80 | 0.89 |
| *Encyonema vulgare* Krammer | ECvulgar | Widely | 0.21 | 3.80 | 0.90 |
| *Navicula bjoernoeyaensis* Metzeltin, Witkowski & Lange-Bertalot | NAbjoern | Circumpolar | 0.26 | 3.61 | 0.85 |
| *Encyonema spitsbergense* Krammer | ECspibaf | Circumpolar | 0.26 | 3.40 | 0.83 |
| *Encyonema reichardtii* (Krammer) Mann | ECreicha | Widely | 0.31 | 3.20 | 0.92 |
| *Gomphonema nathorstii* Foged | Gonathor | Circumpolar | 0.24 | 3.20 | 0.77 |
| *Amphora cf. copulata* (Kützing) Schoeman et Archibald | AMcopucf | Widely | 0.30 | 3.00 | 0.78 |
| *Amphora dusenii* Brunnthaler | Amduseni | Circumpolar | 0.21 | 3.00 | 0.70 |
| *Caloneis fusus* Hamilton & Antoniades | CAfusus | Circumpolar | 0.43 | 2.80 | 0.79 |
| *Stauroneis agrestis* Petersen | SSagrest | Circumpolar | 0.16 | 2.41 | 0.57 |
| *Stauroneis prominula* (Grunow) Hustedt | SSpromin | Circumpolar | 0.13 | 2.41 | 0.57 |
| *Caloneis* spA | CAsp_A | Circumpolar | 0.36 | 2.40 | 0.71 |
| *Eunotia scandiorussica* M.Kulikovskiy, H.Lange-Bertalot, S.Genkal & A.Witkowski | EUscdrus | Circumpolar | 0.24 | 2.40 | 0.63 |
| *Sellaphora rectangularis* (Gregory) Lange-Bertalot et Metzeltin | SFrectan | Unknown | 0.24 | 2.40 | 0.71 |
| *Nitzschia pura* Hustedt | NIpura | Widely | 0.33 | 2.20 | 0.61 |
| *Cymbella af. Subarctica* | CMsubaaf | Unknown | 0.24 | 2.20 | 0.60 |
| *Luticola palaearctica (Hustedt)* Mann | LUpalear | Circumpolar | 0.15 | 2.11 | 0.50 |
| *Placoneis* spA | PC_spA | Unknown | 0.13 | 2.11 | 0.50 |
| *Caloneis holarctica* Kulikovskiy, Lange-Bertalot & Witkowski | CAholart | Circumpolar | 0.27 | 2.00 | 0.54 |
| *Eucocconeis cf. flexella* (Kützing) Cleve | ESflexel | Unknown | 0.27 | 2.00 | 0.54 |
| *Gomphonema subarcticum* Lange-Bertalot & E.Reichardt | GOsubarc | Circumpolar | 0.14 | 2.00 | 0.47 |
| *Boreozonacola hustedtii* Lange-Bertalot, Kulikovskiy & Witkowski | BOhusdet | Circumpolar | 0.11 | 2.00 | 0.47 |
