# Supplementary figures and images for: The structure and diversity of freshwater diatom assemblages from Franz Josef Land Archipelago: a northern outpost for freshwater diatoms
Source: PeerJ. 2016 Feb 18;4:e1705. doi: 10.7717/peerj.1705 (PMC4768701; doi:10.7717/peerj.1705)

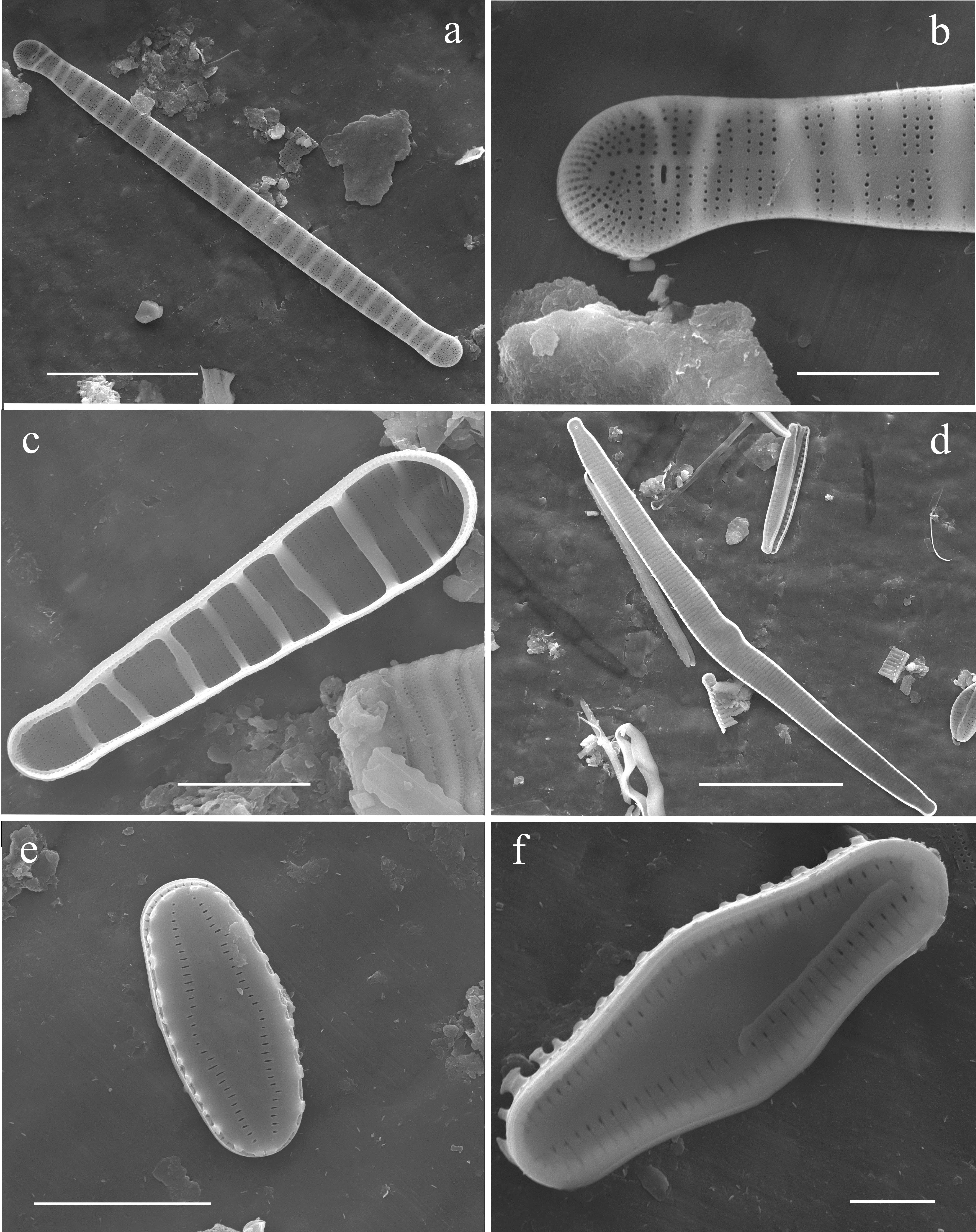

Supplement: Appendix S2 — Figs. (A–B), Diatoma tenuis, external view. Fig. (C), Merdion circulare, internal view. Fig. (D), Hannaea arcus, internal view. Fig. (E), Diadesmis gallica external view. Fig. (F), internal view. Scale bars. Fig. (D) = 20 µm, Fig. (A) = 10 µm, Figs. (C–D) = 5 µm, Figs. (B, F) = 2 µm. [file peerj-04-1705-s002.jpg]

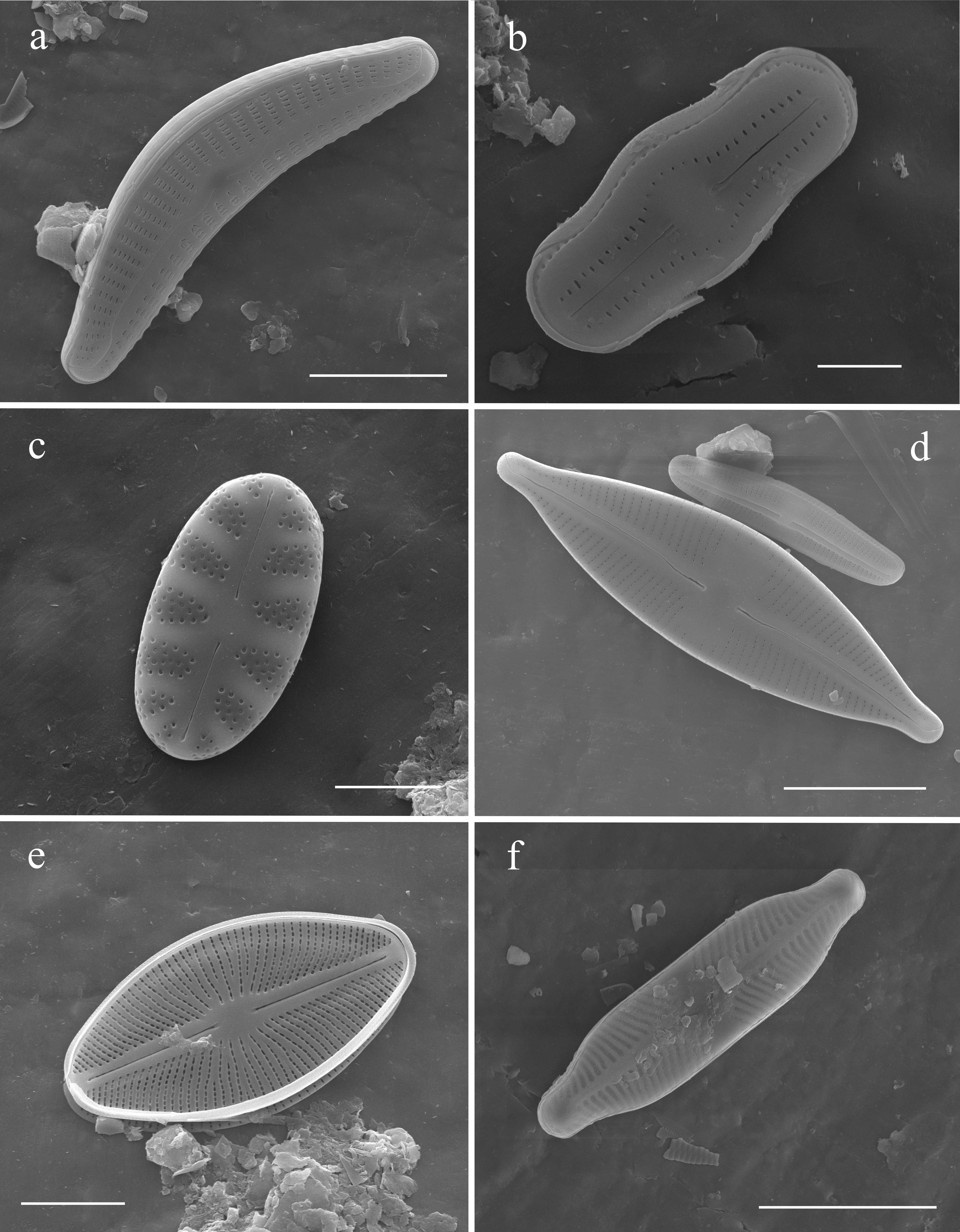

Supplement: Appendix S3 — Fig. (A), Cymbella cleve-eulerae, external view. Fig. (B). Humidophila ingeiiformis, external view. Fig. (C), Hygropetra balfouriana, external view. Fig. (D), Cymbopleura stauroneiformis, external view. Fig. (E), Cavinula cocconeiformis, external view. Fig. (F), Pinnularia biceps, external view. Scale bars. Fig. (F) = 20 µm, Figs. (A, D) = 10 µm, Fig. (E) = 5 µm, Figs. (B–C) = 2 µm. [file peerj-04-1705-s003.jpg]

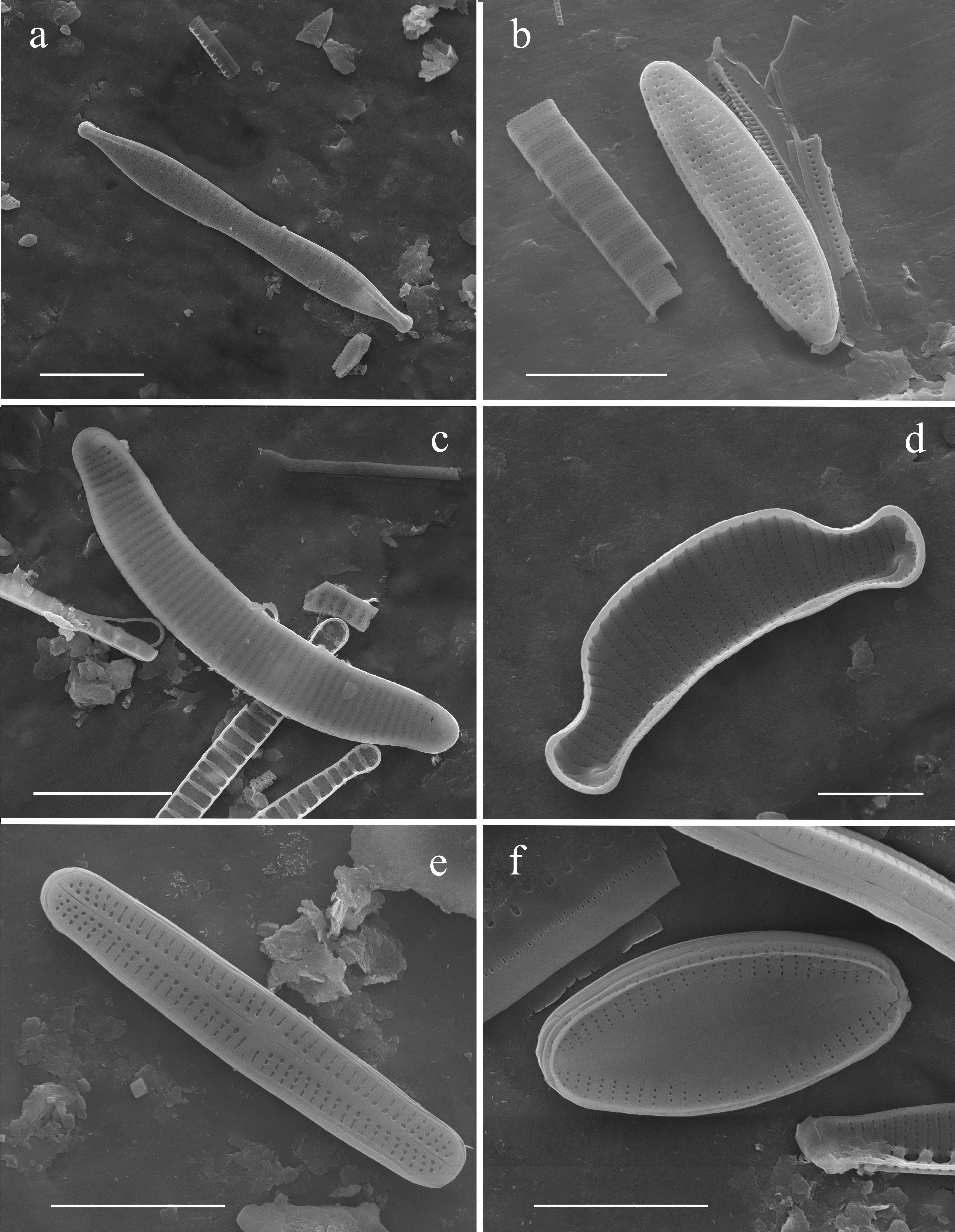

Supplement: Appendix S4 — Plate 3. Fig. (A), Nitzschia homburgiensis, external view, Fig. (B), Nitzschia frustulum, external view, Fig. (C), Eunotia scandiorussica, external view, Fig. (D), Eunotia septentrionalis, internal view. Fig. (E), Achnanthidium sp. (Achnanthidium minutissimum sensu lato), external view, Fig. (F), Psammothidium marginulatum, external view. Scale bars. Figs. (A, C) = 10 μ m, Figs. (B, D, E–F) = 5 µm. [file peerj-04-1705-s004.jpg]
